# A cryptic promoter in the exon of *HKR1* drives expression of a truncated form of Hkr1 in *Saccharomyces cerevisiae*

**DOI:** 10.1371/journal.pone.0314016

**Published:** 2024-11-21

**Authors:** Toshihiro Kondo, Yuna Hosokawa, Ryotaro Ozawa, Shin Kasahara

**Affiliations:** Food Microbiology Unit, Miyagi University School of Food Industrial Sciences, Sendai, Japan; CNR, ITALY

## Abstract

*Hansenula mrakii* killer toxin resistant gene 1 (*HKR1*) is an intronless, single-exon gene that encodes Hkr1, the signaling mucin of the budding yeast *Saccharomyces cerevisiae*. *HKR1* overexpression confers *S*. *cerevisiae* cells with resistance to the HM-1 killer toxin produced by the killer yeast *Hansenula mrakii* (currently known as *Cyberlindnera mrakii*). Hkr1 comprises multiple functional domains and participates in several signal transduction pathways, including the high-osmolarity glycerol (HOG) pathway, the cell wall integrity (CWI) mitogen-activated protein (MAP) kinase pathway, and the filamentation MAP kinase pathway; Hkr1 also controls bud-site selection. In this study, we identified a cryptic promoter in the *HKR1* exon that regulates the transcription of a shorter transcript encoding a truncated form of Hkr1. This shorter protein still conferred resistance to the HM-1 killer toxin, suggesting that this cryptic promoter helps carry out Hkr1-mediated signal transduction efficiently by producing a specific Hkr1 domain with functions as a signaling messenger. Notably, reporter assays using the fluorescent protein gene *mUkG1* and the β-galactosidase gene *lacZ* revealed that the transcriptional activity of this cryptic promoter was modulated by its upstream sequence within the single exon. Hkr1 thus differs from other signaling mucins, whose active C-terminal fragments are generated by post-translational processing, whereas the active C-terminal fragment of Hkr1 is generated by transcription from the exonic promoter. These findings describe a previously unknown example of functional diversification from a single gene, especially for a gene encoding a multidomain, multifunctional protein such as Hkr1.

## Introduction

The budding yeast *Saccharomyces cerevisiae* is an important microorganism most widely used in the fermentation industry, particularly for the production of bread, alcoholic beverages, food seasonings, health foods, and even biofuels. Yeast cells are continuously exposed to various stresses under artificially controlled industrial conditions, but also in their natural environment. These stressors include high and low temperatures, limited water availability, changes in osmotic pressure, nutritional deficiency, and the presence of cytotoxic substances or competing microorganisms.

*Hansenula mrakii* killer toxin resistant protein 1 (Hkr1), a signaling mucin of *S*. *cerevisiae*, is a heavily glycosylated type I transmembrane protein consisting of 1,802 amino acids. Hkr1 is an osmosensor that initiates the high-osmolarity glycerol (HOG) pathway in response to high-osmolarity conditions [1–3]. The extracellular region of Hkr1 accounts for over 80% of the entire protein and contains a signal peptide and a tandem repeat domain with a high content of serine, threonine, and proline amino acid residues unique to mucin family proteins. The Hkr1-Msb2 (multicopy suppression of a budding defect 2) homology domain, which is essential for the activation of the HOG pathway, is adjacent to the transmembrane domain [1]. On the cytoplasmic side, Hkr1 contains calcium-binding EF hand and leucine zipper motifs, suggesting a role in signal transduction and transcriptional control. *HKR1* was first identified as a gene whose overexpression in *S*. *cerevisiae* cells conferred resistance to the HM-1 killer toxin (abbreviated as HM-1) produced by the killer yeast *Hansenula mrakii* (currently known as *Cyberlindnera mrakii*) [4, 5]. The exact mechanism behind the cytocidal activity of HM-1 is not clear, but previous studies have suggested that HM-1 inhibits the biosynthesis of cell wall β-glucan [6] and likely influences the cell wall integrity (CWI) mitogen-activated protein (MAP) kinase pathway [7]. In fact, Hkr1 is now recognized as a key component of the CWI pathway [7, 8]. It was thought that the HOG and CWI MAP kinase pathways were independent, but later studies revealed that the two pathways, both of which rely on Hkr1, are positively coordinated to regulate stress responses, making Hkr1 a crucial factor in stress sensing and acclimation. Actually, *S*. *cerevisiae* cells require Hkr1 and elements of the synthetic high-osmolarity-sensitive 1 (SHO1) branch of the HOG pathway to respond to cell wall damage induced by Zymolyase [7, 9–11]. Moreover, Hkr1 regulates the filamentation MAP kinase pathway [12] and is involved in bud-site selection, based on the phenotype associated with a mutation in the sequence encoding its C-terminal region, resulting in aberrant morphology [5]. Thus, these functional and structural characteristics support the notion that Hkr1 is a multifunctional protein consisting of multiple domains.

Generally, membrane-associated mucins function as sensors of the external environment when ligand molecules bind to their extracellular domain or when their conformation changes in response to external stimuli such as a change in osmolarity, pH, or the concentration of chemical substances. These signals are then transmitted inside the cell through post-translational modifications of their intracellular domain, commonly called the cytoplasmic tail (CT). Phosphorylation and proteolytic cleavage are typical post-translational modifications observed in mammalian mucins. MUC1, a well characterized transmembrane mucin, associates with signaling molecules including β-catenin, γ-catenin, p53, and estrogen receptor α. The CT of MUC1 (MUC1CT) is critical in the downstream cellular signaling cascades via direct physical interaction. MUC1CT also binds to transcription factors, and the resulting complex translocates to the nucleus, where it regulates transcription of target genes [13–16].

Msb2 of *S*. *cerevisiae*, a sister mucin of Hkr1, is often compared to MUC1 and other mammalian mucins as a prototypical example of a signaling mucin from eukaryotic cells. Indeed, these mucins share structural and functional features and undergo similar post-translational processing [17]. Msb2 was originally identified as a multicopy suppressor of the bud emergence defect seen in *S*. *cerevisiae* defective in cell division cycle 24 (*cdc24*) cells when overexpressed [18]. Later studies revealed that Msb2 plays an important role in the SHO1 branch of the HOG pathway as an osmosensor [1, 2]. Msb2 was also reported to interact with signaling molecules, such as the small GTP-binding protein Cdc42 and the transmembrane osmosensor Sho1, to promote their function within the filamentous growth (FG) pathway [19]. Furthermore, the HOG pathway modulates the FG pathway, with Msb2 and Hkr1 in the HOG pathway controlling the FG pathway in different ways [12]. Thus, Hkr1 and Msb2 not only share structural characteristics, but also display some overlap in their functions. Like transmembrane mucins in mammalian cells, Msb2 undergoes proteolytic cleavage in its active form [15, 20, 21], which is catalyzed by the aspartyl protease yapsin 1 (Yap1) [20]. Despite the wealth of information about Hkr1, its functions in the HOG and CWI MAP kinase pathways, and its interactions with surrounding factors [1–3, 22, 23], there is currently limited information about the post-translational regulation.

When first isolated as the gene conferring resistance to HM-1, the *HKR1* clone was promoterless and truncated, starting in the middle of the single exon, but was nevertheless clearly sufficient to confer resistance [4]. This initial observation raised questions about the contribution of full-length *HKR1* to HM-1 resistance. Transcription typically begins from the promoter region located upstream of the 5′ end of the gene and terminates downstream of the 3′ end. The functionality of a 5′-truncated *HKR1* transcript suggests that the upstream DNA sequence may exhibit promoter activity in addition to, or instead of, encoding the N-terminal portion of endogenous Hkr1.

In our plasmid-based study, we demonstrate that a cryptic promoter exists in the *HKR1* exon (hereinafter referred to as an exonic promoter), with the ATG codon immediately downstream and corresponding to the methionine residue at the position 1137 (Met-1137) in full-length Hkr1 functioning as an internal translation initiation site. In reporter assays using the fluorescent protein gene *mUkG1* and the β-galactosidase gene *lacZ*, we found that the activity of this cryptic promoter was repressed by its upstream sequence within the same exon, but when NaCl or sorbitol was added to the culture to create hyperosmotic conditions, we also observed a tendency for the number of fluorescence-positive cells to increase. Our findings offer another noteworthy example of diverse manners by which a single gene can encode multiple functional proteins, especially for a gene encoding a multidomain, multifunctional protein such as Hkr1.

## Materials and methods

### Yeast strains, chemicals, and culture media

Throughout this study, *Saccharomyces cerevisiae* strain A451 (ATCC 200589, *MAT*α *can1 leu2 trp1 ura3 aro7*) was used for genetic manipulations and as the control strain. *Hansenula mrakii* (synonym *Cyberlindnera mrakii*, ATCC10743) was used for the production of HM-1.

All chemicals were purchased through Nacalai Tesque; the ingredients for culture medium were obtained from BD Difco.

YPD medium (1% [w/v] yeast extract, 2% [w/v] peptone, 2% [w/v] glucose) was used as the standard medium for general culture, while the synthetic minimal medium (0.17% [w/v] yeast nitrogen base without amino acids and ammonium sulfate, 0.5% [w/v] ammonium sulfate, 2% [w/v] glucose supplemented with required amino acids, pH 6.5) was used for selective culture under leucine or uracil auxotrophy. For the experiments in which the *GAL1* promoter (pro*GAL1*) was used to drive gene expression, the galactose (2% [w/v])-containing minimal medium was used. To prepare solid media, agar was added to 2% (w/v). To prepare HM-1, *H*. *mrakii* was cultured in the yeast nitrogen base (YNB) medium (0.17% [w/v] yeast nitrogen base, 2% [w/v] glucose).

### Genetic methods

Standard methods of genetic manipulation were used [24]. Restriction enzymes and a DNA ligase for cloning were purchased through Takara Bio. The DNA sequences of *HKR1* used in this study were isolated and manipulated by PCR-based methods using genomic DNA of *S*. *cerevisiae* A451 as template. For PCR, PrimeSTAR GXL DNA Polymerase (Takara Bio) was used with the primers listed in S1 Table. The primers were synthesized by Nihon Gene Research Laboratories. The PCR amplicons cloned in the plasmid vectors were all subjected to Sanger sequencing and confirmed to harbor no mutations. To determine the region responsible for resistance to HM-1, various lengths of the *HKR1* sequence were subcloned into the plasmid pYES2 (Invitrogen) to be expressed from the inducible *GAL1* promoter (pro*GAL1*). The plasmids used in this study are listed in S2 Table.

### Preparation of the HM-1 killer toxin

HM-1 was prepared from a culture filtrate of *H*. *mrakii*. Precultured *H*. *mrakii* was used to inoculate YNB medium; the resulting culture was incubated at 30°C for 48 h with agitation at 100 rpm. Cells were removed by centrifugation (8,000×*g*, 10 min, 4°C) followed by filtration through a 0.45-μm-pore-size filter (Nalgene). The filtrate was dialyzed against distilled water before being lyophilized with an FDM-1000 freeze dryer (EYELA). The powdered HM-1 was resuspended in distilled water and filter-sterilized with a 0.45-μm-pore-size filter. Since the *H*. *mrakii* ATCC10743 strain exclusively secrets HM-1 into the culture, no further purification steps were applied. The concentration of HM-1 that killed 75% or 50% of all *S*. *cerevisiae* A451 cells was determined by serial dilution.

### Assay for resistance to the HM-1 killer toxin

To test HM-1 resistance in liquid culture, *S*. *cerevisiae* transformants were inoculated into the synthetic minimal medium containing HM-1 at the 75% lethal dose and incubated at 30°C for 48 h with agitation at 100 rpm. After 48 h, the optical density at 600 nm (OD_600_) of each culture was measured. The same medium without HM-1 was used as a control.

For the plate assay, *S*. *cerevisiae* transformants were sparsely and uniformly plated onto the synthetic minimal agar medium. *H*. *mrakii* was then spotted at the center of each plate and incubated at 30°C. After 2–3 days, the formation of a clear zone around the spotted *H*. *mrakii* cells was observed.

### Construction of reporters based on the fluorescent protein mUkG1

A plasmid carrying the full-length *mUkG1* sequence encoding the fluorescent protein mUkG1 (monomeric Umikinoko-Green 1) from the soft coral *Sarcophyton* sp. was purchased from Medical & Biological Laboratories. In this study, mUkG1 was used instead of the conventional green fluorescent protein (GFP) from *Aequorea victoria* because of its more advantageous codon usage for expression in yeast cells and the stability of the produced protein compared to GFP [25, 26]. For the reporter assays, the full-length *mUkG1* sequence was ligated to the 160-bp region starting from the *Hin*dIII site at the nucleotide position #3249 (using the A of the original ATG codon as position #1) in frame with *HKR1*, with various lengths of the *HKR1* exon. The original *HKR1* terminator (*HKR1*ter) was used to terminate transcription. The *S*. *cerevisiae* alcohol dehydrogenase gene 1 (*ADH1*) promoter (pro*ADH1*) was used as a positive control in combination with the *ADH1* terminator (*ADH1*ter). These reporter constructs used the 2-micron multicopy plasmid as backbone and were transformed into *S*. *cerevisiae* A451 cells.

### Fluorescence microscopy

Transformed *S*. *cerevisiae* cells were grown in the synthetic defined medium at 30°C. When using a culture medium not containing added NaCl or sorbitol, cells were cultured until OD_600_ reached 0.8–1.0. In experiments with medium containing added NaCl or sorbitol, the cells were grown in the presence of 250 mM NaCl or 1 M sorbitol for the same duration as control cultures or until OD_600_ reached 0.8–1.0 (typically about 18 h). Cells were then collected by centrifugation (17,800×*g*, 3 min, 20°C), and autofluorescent dead cells were removed via cell sorting on a CytoFLEX SRT (Beckman Coulter) set at 488 nm for excitation with 525/40 and 690/50 band-pass filters as needed. Fluorescence observations of the mUkG1-expressing cells were performed using an Axio Imager.A1 fluorescence microscope system with an Apochromat 40× objective lens (Carl Zeiss). Photographs were taken with an AxioCam MRm camera. The software AxioVision Rel. 4.8 was used for image acquisition and analysis.

### Flow cytometry

Transformed *S*. *cerevisiae* cells were grown in the synthetic defined medium at 30°C. Treatment with NaCl or sorbitol was carried out under the same conditions as those used for fluorescence microscopy. For cells grown under the influence of HM-1, the transformants were cultured in the medium containing the 50% lethal concentration of HM-1 for 18 h. Cells were collected by centrifugation (17,800×*g*, 3 min, 20°C), washed twice with distilled water, and resuspended in distilled water. A flow cytometer CytoFLEX (Beckman Coulter) was used at 488 nm for excitation with a 525/40 band-pass filter. Approximately 10,000 cells were analyzed for each sample, and CytExpert Version 2.5 software (Beckman Coulter) was used for data acquisition and analysis.

### β-Galactosidase assay

For the quantitative reporter assays, the *Escherichia coli* β-galactosidase gene *lacZ* and a centromeric plasmid were used. The drug resistance gene *AUR1-C* of the yeast centromeric plasmid vector pAUR112 (Takara Bio) was replaced with *lacZ*-reporter cassettes ligated to various lengths of the *HKR1* exon, then introduced into *S*. *cerevisiae* A451 cells. The original *HKR1* terminator (*HKR1*ter) was used to terminate transcription. Measurements of β-galactosidase activity were performed using *o*-nitrophenyl β-D-galactopyranoside (ONPG) as a substrate, and Miller units were calculated [27]. Transformed *S*. *cerevisiae* cells were cultured in the synthetic defined medium until OD_600_ reached around 0.6 before being collected by centrifugation (17,800×*g*, 1 min, 20°C). Cells were then broken by repeated freeze-thaw cycles. The reaction time for color development was 1 h for all measurements.

### 5′-RACE for the determination of transcription start site

5′ Rapid amplification of cDNA ends (5′-RACE) was performed with a 5′-Full RACE Core Set (Takara Bio). The transformant harboring the plasmid with the sequence of *HKR1* from the nucleotide position #3249 to the *HKR1* terminator (2,598 bp), #2997 to the terminator (2,850 bp), or #2614 to the terminator (3,233 bp) was used. Total RNA was extracted with TRIzol Reagent (Invitrogen) from each transformant and reverse transcribed. Primers used for the reaction are listed in S1 Table.

### Quantitative PCR

Quantitative PCR for the evaluation of copy number stability of the 2-micron multicopy plasmids was performed with TB Green *Premix Ex Taq* II (Takara Bio) and a real-time qTOWER^3^ G thermal cycler (Analytik Jena). Total DNA was extracted from exponentially growing cells by the freeze-thaw method [28] with slight modifications. Briefly, harvested cells were broken by repeated freeze-thawing cycles with liquid nitrogen and boiling water, followed by phenol-chloroform extraction to recover DNA. An equal amount (1 ng) of total DNA between the samples was used as template for quantitative PCR. The reporter gene *mUkG1* contained in the plasmids was used as the target. To normalize the quantification, the endogenous *HKR1* sequence, which is not contained in the plasmids, was targeted. The primers used for quantitative PCR are listed in S1 Table.

## Results

### Identification of a region responsible for HM-1 killer toxin resistance and a possible internal translation initiation site in the exon of *HKR1*

**Fig 1:**
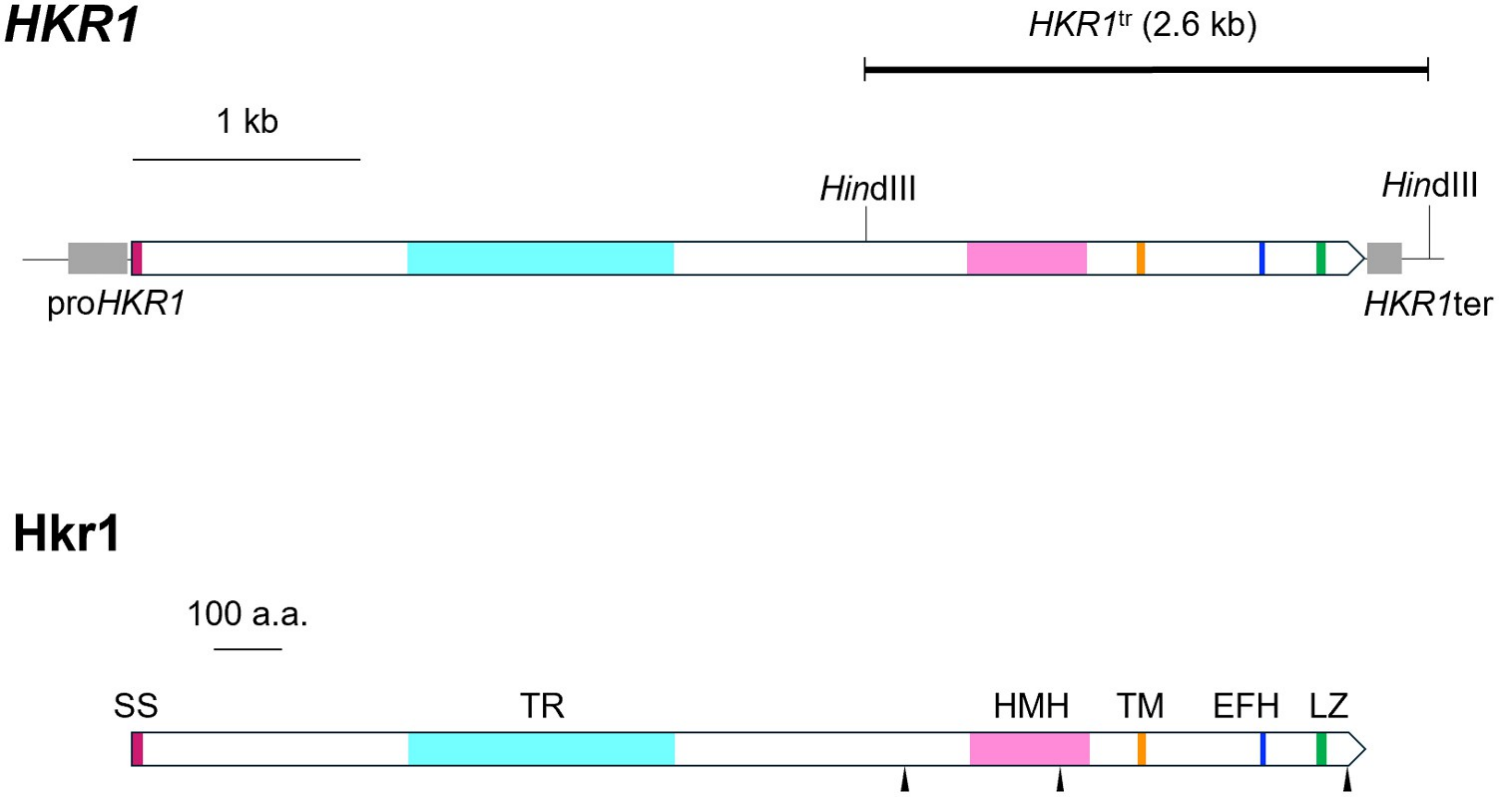
Diagrams of the *S*. *cerevisiae HKR1* locus and the protein Hkr1 encoded by *HKR1*. Diagram of the *HKR1* locus, consisting of a 5.4-kb open reading frame encoding a mucin-like type I transmembrane protein Hkr1 with its original promoter (pro*HKR1*) and terminator (*HKR1*ter). *HKR1*^tr^ designates the 2.6 kb-truncated portion of *HKR1* with the terminator used for HM-1 resistance assay, flanked by two *Hin*dIII restriction sites. The N-terminal signal peptide sequence of Hkr1 (SS) is shown in dark red, and the extracellular tandem repeat domain (TR) commonly found in most mucins is in light blue. The Hkr1-Msb2 homology domain (HMH) is shown in pink, the transmembrane domain (TM) is indicated in orange, and the EF hand (EFH) and the leucine zipper (LZ) regions are shown in blue and green, respectively. Three internal methionine residues, Met-1137, Met-1368, and Met-1788, found in the same frame as Hkr1 that could serve as internal translation initiation sites are indicated by wedges.

*HKR1* encodes the large transmembrane protein Hkr1, and like many other genes of *S*. *cerevisiae*, it is an intronless gene (Fig 1). The 3′ portion of *HKR1* (*HKR1*^tr^, a 2.6-kb *Hin*dIII–*Hin*dIII fragment, shown in Fig 1) was identified as being sufficient to confer resistance against HM-1 [4], which was surprising because this fragment did not encode the entire Hkr1 protein. To confirm this result, we cloned this 2.6-kb *HKR1*^tr^ fragment (sequence I in S1A Fig) downstream of the *GAL1* promoter (pro*GAL1*) in the pYES2 backbone (construct I, S1A Fig) and transformed *S*. *cerevisiae* A451 cells with the resulting construct to test their resistance to HM-1. When expressed from the *GAL1* promoter present in pYES2, this *HKR1*^tr^ fragment indeed conferred resistance to HM-1 in an assay using liquid culture (S1B Fig), suggesting that this fragment contains sequences important for this process. We also tested three additional constructs carrying shorter fragments (S1A Fig): construct II, containing the sequence starting immediately downstream of the ATG codon encoding Met-1137 in full-length Hkr1 (sequence II); construct III, corresponding to the 1,406-bp sequence from the nucleotide position #4441 to the terminator region (sequence III); and construct IV, harboring the sequence from the nucleotide position #4525 to the terminator region (sequence IV). Notably, none of these additional constructs led to resistance to HM-1 in a resistance assay (S1B Fig). Of the four partial *HKR1* sequences tested, only sequence I contains the ATG codon for Met-1137, from which translation may be initiated. The first methionine residue in sequence II is Met-1368, while sequences III and IV are not predicted to encode a protein larger than 15 amino acids in the same open reading frame (S1B Fig). In addition, the protein predicted to be translated from sequence I would include the Hkr1-Msb2 homology domain, which is essential for Hkr1 function in the HOG pathway [1], whereas sequences III and IV would not; the protein predicted to be translated from sequence II may contain the last 60 amino acids of the Hkr1-Msb2 homology domain, out of 218. In the other two reading frames in sequences I–IV, several ATG codons are found, but frequent appearance of stop codons makes it impossible to encode proteins longer than 60 amino acids.

The above result suggested the possibility that the ATG codon starting at the position #3409 in *HKR1* and corresponding to Met-1137 might be used as a translation initiation site for the truncated form of Hkr1 (Hkr1^tr^), which we tested next.

### A truncated Hkr1 initiated from an internal methionine confers resistance to the HM-1 killer toxin

**Fig 2:**
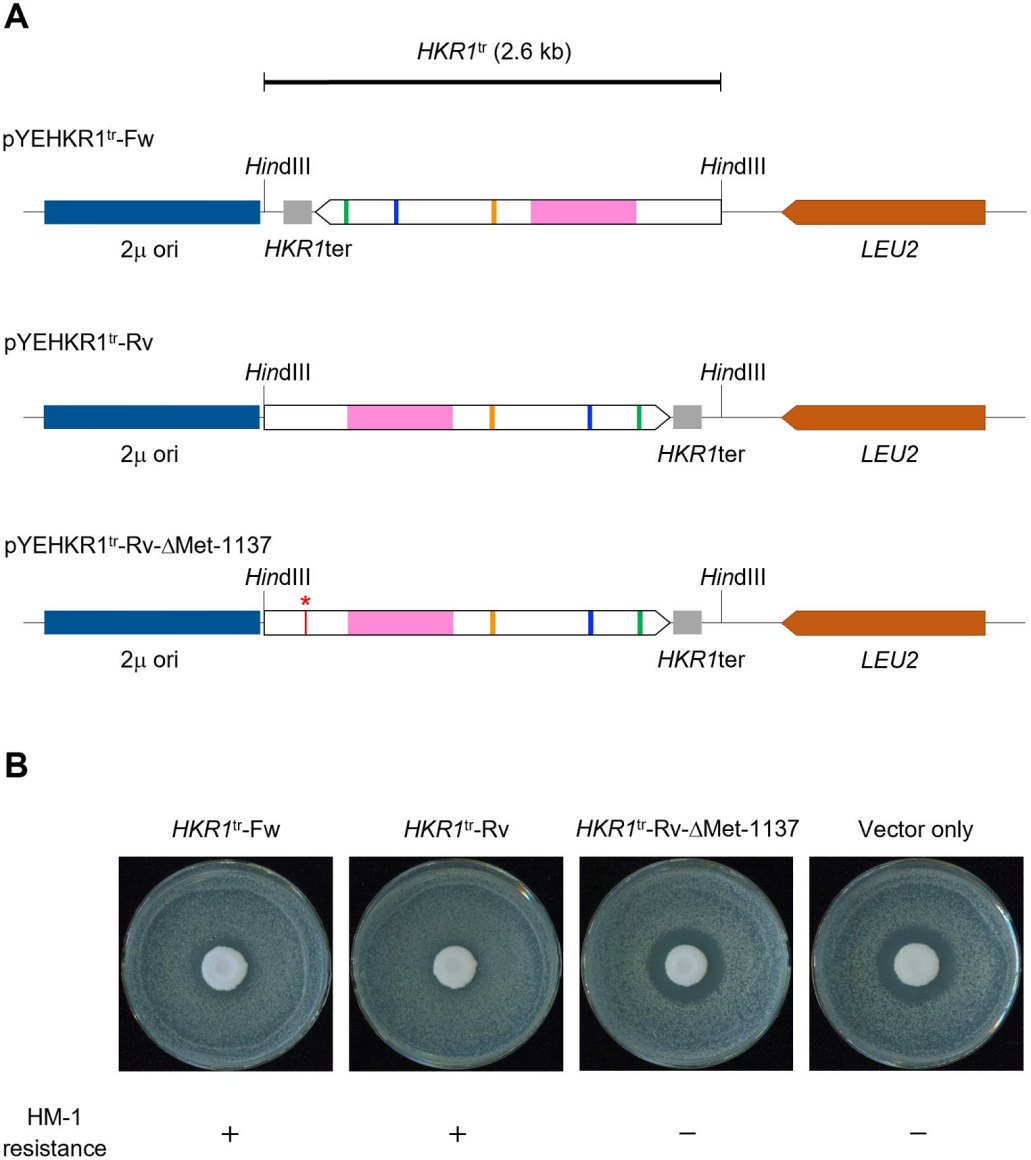
Resistance to the HM-1 killer toxin of transformants expressing a truncated version of Hkr1 and importance of Met-1137 for the acquisition of resistance. **(A)** Constructs of the plasmids harboring the 2.6-kb fragment of *HKR1* (*HKR1*^tr^) with its original terminator (*HKR1*ter) introduced into *S*. *cerevisiae* A451 cells on the 2-micron multicopy vector, in either the forward or reverse direction relative to the *LEU2* marker (pYEHKR1^tr^-Fw or pYEHKR1^tr^-Rv). The construct of the plasmid pYEHKR1^tr^-Rv-ΔMet-1137, harboring *HKR1*^tr^-ΔMet-1137 in the 2-micron multicopy vector, with the ATG codon of Met-1137 replaced with the stop codon TAG. The plasmid vector without the *HKR1*^tr^ insert (Vector only) served as a control. The asterisk and the line in red in the pYEHKR1^tr^-Rv-ΔMet-1137 construct indicate the position of the ATG codon replaced with the stop codon TAG. **(B)** Representative photographs of transformants carrying *HKR1*^tr^ showing resistance to HM-1 regardless of the direction in which *HKR1*^tr^ was inserted into the vector while the transformant carrying the plasmid pYEHKR1^tr^-Rv-ΔMet-1137 showed sensitivity to HM-1. *H*. *mrakii* cells producing HM-1 were spotted over the lawn of the *S*. *cerevisiae* transformants in the center of the plates. The formation of a clear zone around the *H*. *mrakii* spot indicates sensitivity to HM-1.

Following the initial isolation of a truncated *HKR1* clone, we assumed that transcription was being carried out by some sequence from the plasmid YEp213, possibly by read-through transcription [4]. To test this possibility, we cloned *HKR1*^tr^ in either orientation into the 2-micron multicopy plasmid (pYEHKR1^tr^-Fw/-Rv in Fig 2A), which revealed resistance to HM-1 in both cases, as evidenced by a plate assay (Fig 2B). We concluded that transcriptional read-through is unlikely to explain the resistance observed when *HKR1*^tr^ is expressed from the plasmid YEp213.

As the introduction of construct I conferred resistance to HM-1, but not that of construct II in S1 Fig, we wondered if a truncated protein starting at Met-1137 might be responsible for this resistance. Accordingly, we replaced the ATG codon encoding Met-1137 with TAG and examined the effect of this mutation on resistance to HM-1 (pYEHKR1^tr^-Rv-ΔMet-1137 in Fig 2A). Importantly, introducing the construct pYEHKR1^tr^-Rv-ΔMet-1137 into *S*. *cerevisiae* did not confer resistance to HM-1, suggesting that the translation of Hkr1^tr^ from Met-1137 is necessary for the observed HM-1 resistance (Fig 2B).

Taken together, we conclude that sequence I contains a promoter or a promoter-like region that causes autonomous transcription, producing a transcript from which translation of Hkr1^tr^ is initiated at Met-1137.

### Reporter gene-based characterization of the exonic *HKR1* promoter

**Fig 3:**
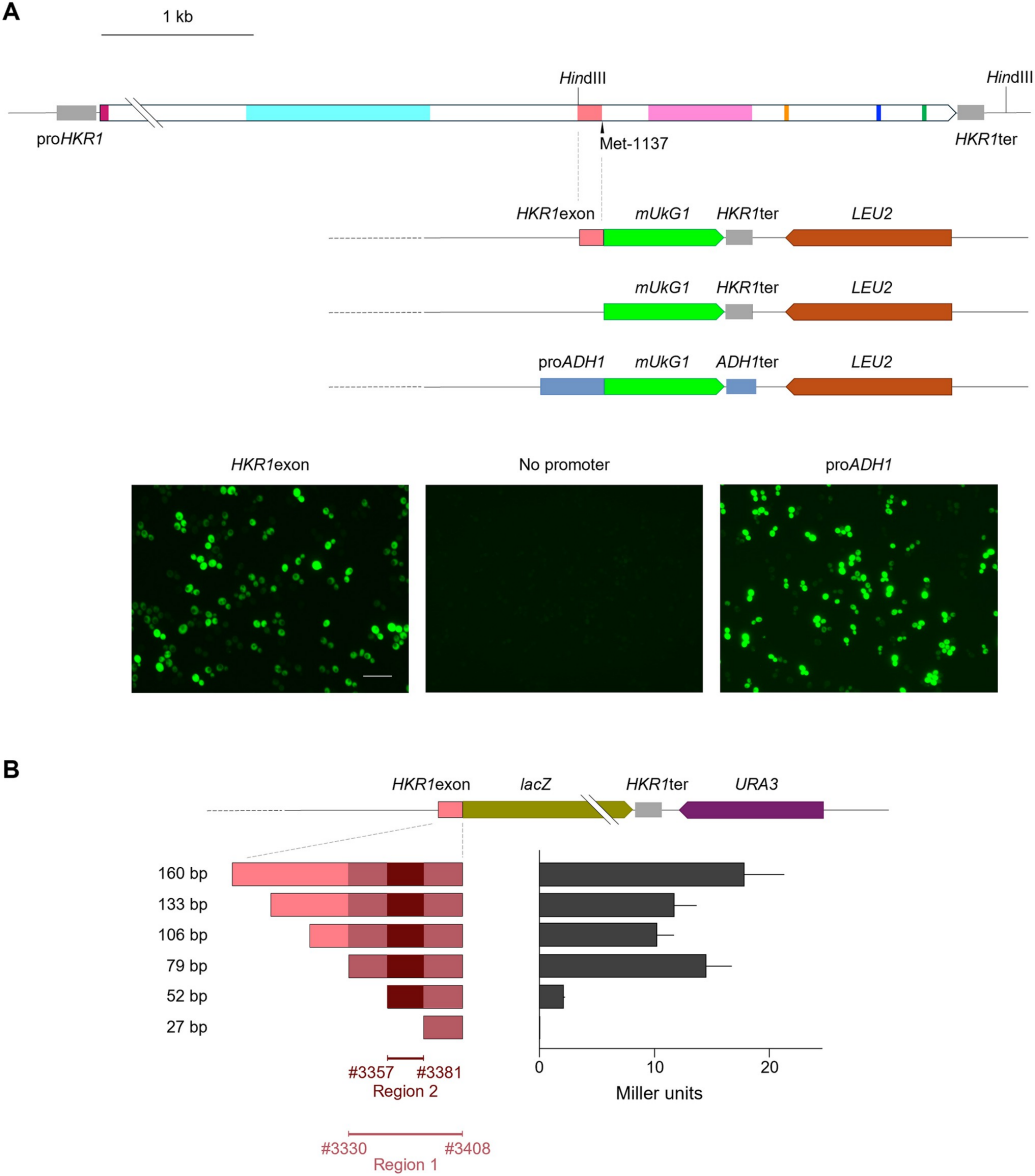
Promoter activity exhibited by the *HKR1* exon and its 5′ deletion series in *S*. *cerevisiae* cells. **(A)** Diagrams of the reporter construct harboring the 160-bp fragment from the *HKR1* exon (from the nucleotide position #3249 to #3408, *HKR1*exon) cloned upstream of the reporter gene *mUkG1* encoding the fluorescent protein mUkG1 in the multicopy plasmid vector, resulting in pYE-exon160UkG1. The plasmid was then introduced into *S*. *cerevisiae* A451 cells. The *S*. *cerevisiae ADH1* promoter (pro*ADH1*) was used as a positive control (pYE-ADHUkG1). Fluorescence of exponentially growing transformants carrying pYE-exon160UkG1 (*HKR1*exon), a promoterless *mUkG1* construct (No promoter), or pYE-ADHUkG1 (pro*ADH1*) was observed and photographed under a fluorescence microscope. The scale bar indicates a length of 20 μm. **(B)** Construct harboring 160-bp *HKR1*exon or its 5′ deletion series variants ligated upstream of the reporter gene *lacZ*. A resulting cassette was inserted into the YCp-based centromeric plasmid vector. β-Galactosidase activity was measured with *o*-nitrophenyl β-D-galactopyranoside (ONPG) as a substrate and is shown as Miller units [27]. Region 1 is a sequence important for sufficient transcription, and Region 2 is necessary for basal transcription. Eight independent primary transformants were tested for each construct. Mean values are given with standard deviation.

We showed above that *HKR1*^tr^ can be transcribed from the region present in sequence I in S1A Fig and is sufficient to confer *S*. *cerevisiae* cells with resistance to HM-1. Upstream of the ATG codon corresponding to Met-1137, sequence I also contains the 160-bp region of the exon that may function as a promoter. To test this idea, we cloned the fluorescent protein gene *mUkG1* downstream of this 160-bp sequence between the *Hin*dIII site and the ATG codon coding for Met-1137 on a multicopy vector (Fig 3A) and introduced the resulting plasmid into *S*. *cerevisiae* A451 cells. We also cloned the same 160-bp exonic sequence upstream of the β-galactosidase gene *lacZ* on a centromeric plasmid to ensure plasmid copy number stability for a quantitative assessment of promoter strength (Fig 3B).

In fluorescence microscopy observations, the transformants harboring *mUkG1* driven by the 160-bp exonic fragment (*HKR1*exon) showed strong fluorescence, as did *S*. *cerevisiae* cells carrying a construct where *mUkG1* was expressed from the *ADH1* promoter (pro*ADH1*). Thus, the 160-bp sequence acts as a promoter from which a functional mUkG1 can be produced (Fig 3A). Similarly, the same 160-bp sequence drove expression of *lacZ* and resulted in considerable β-galactosidase activity (Fig 3B).

We wondered if the 160-bp sequence represented a partial exonic promoter, as its length was dictated by the *Hin*dIII restriction sites. Accordingly, we performed a 5′ deletion promoter resection series or extended the sequence further upstream to assess the consequences of having a shorter or longer exon fragment on promoter activity. Truncating the 160-bp exonic sequence at its 5′ end only resulted in strongly decreased β-galactosidase activity following the deletion of 108 bp downstream of the *Hin*dIII site, with almost no detectable activity upon the deletion of 133 bp (Fig 3B). The result of this experiment indicated that the 79-bp sequence from the nucleotide position #3330 to #3408 (Region 1 in Fig 3B) likely contains a sequence important for sufficient transcription, while the sequence from #3357 to #3381 (Region 2 in Fig 3B) is necessary for basal transcription.

**Fig 4:**
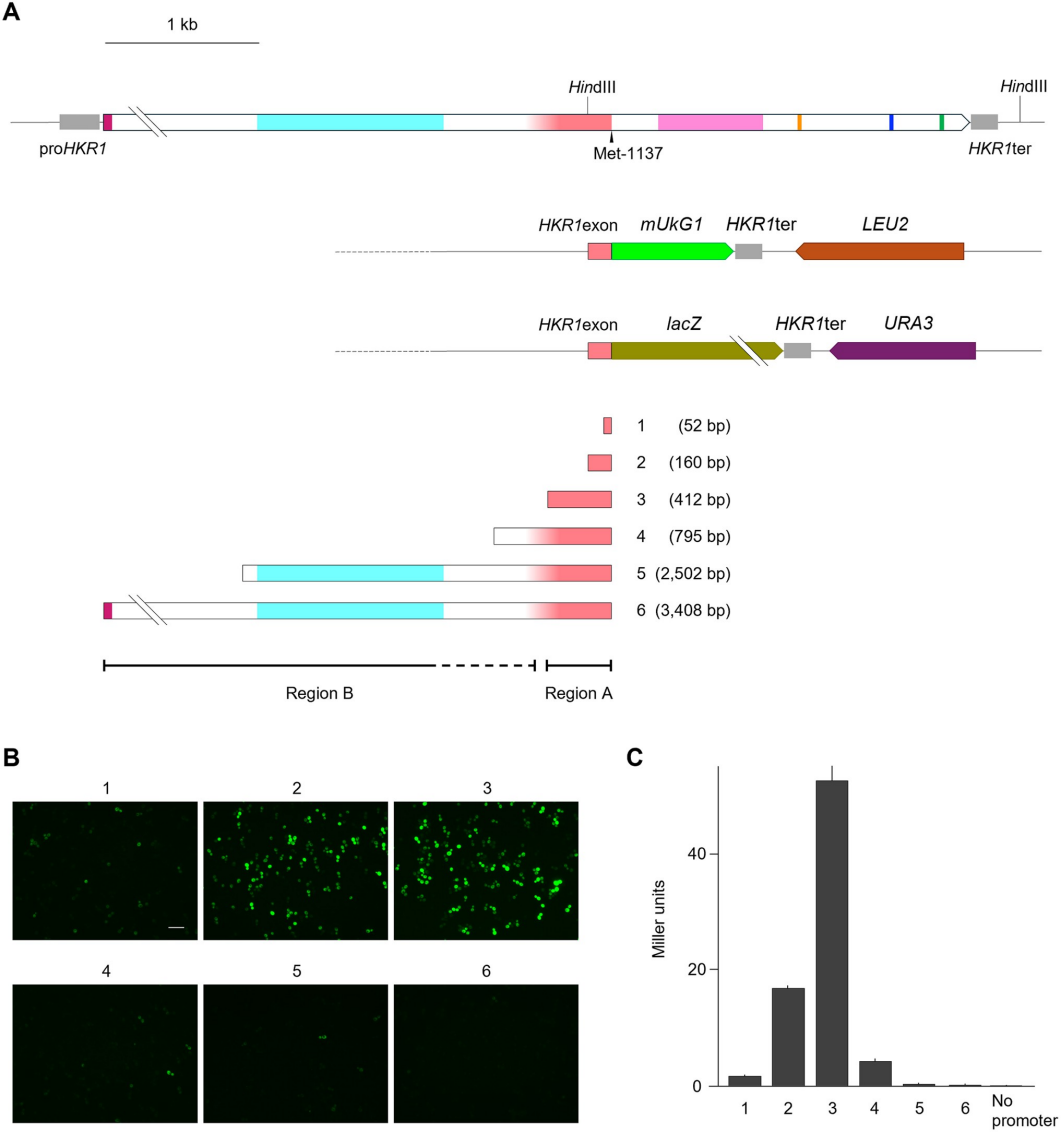
A sequence upstream of the exonic *HKR1* promoter represses its transcription in *S*. *cerevisiae* cells. **(A)** Diagrams of the reporter constructs in which the fluorescent protein gene *mUkG1* or the β-galactosidase gene *lacZ* is driven by the *HKR1* exon fragment 1, 2, 3, 4, 5, or 6. Region A is the core sequence of the exonic promoter, while Region B acts as a silencer-like sequence that negatively regulates its transcriptional activity. **(B)** Fluorescence examination of exponentially growing transformants carrying the indicated constructs observed with a fluorescence microscope. The scale bar indicates a length of 20 μm. **(C)** Quantitative measurement of β-galactosidase activity from exponentially growing cells harboring the constructs shown in **(A)**. The transformant carrying promoterless *lacZ* (No promoter) served as a negative control. β-Galactosidase activity is calculated as Miller units [27]. Eight independent primary transformants were tested for each construct, and means with standard deviation are shown.

Conversely, when we extended the exonic promoter sequence on the 5′ side of the 160-bp fragment (Fig 4A), we detected stronger fluorescence and a 3-fold increase in β-galactosidase activity with a 412-bp fragment upstream of the ATG codon corresponding to Met-1137 (Fig 4B and 4C). As an important control, we checked the copy number of each plasmid containing *mUkG1* by quantitative PCR, which showed that the measured changes in fluorescence intensity derived from the reporters were not due to fluctuations in plasmid copy number (S2 Fig). Therefore, the 160-bp sequence is unlikely to represent the full promoter region; rather, the sequence after the nucleotide position #3000 defines the core exonic promoter of *HKR1*^tr^ (Region A in Fig 4A).

If *HKR1*^tr^ is transcribed from an exonic promoter that overlaps with the *HKR1* single exon, we should be able to identify the transcription start site by conducting a 5′-RACE assay. In this experiment, we used the transformants harboring the plasmid with the sequence of *HKR1* from the nucleotide position #3249 to the *HKR1* terminator (2,598 bp, pYEHKR1-Rv-3249), #2997 to the terminator (2,850 bp, pYEHKR1-Rv-2997), or #2614 to the terminator (3,233 bp, pYEHKR1-Rv-2614), in which the exonic sequence 2, 3, or 4 in Fig 4A acts as a promoter to express Hkr1^tr^, respectively (S3 Fig). As a result, we obtained three putative transcription start sites mapping upstream of the ATG codon corresponding to Met-1137: one transcription start site at the nucleotide position #3075, one at #3361, and one at #3383 (S3 Fig). As the *Hin*dIII restriction site is located at #3249, the two transcription start sites at the positions #3361 and #3383 may initiate transcription of *HKR1*^tr^, although it is possible that the site at #3075 may also be a functional transcription start site depending on the length of the promoter region used in our plasmid-based experiment.

### Involvement of the longer upstream sequence of the exonic *HKR1* promoter within the same exon in transcriptional regulation

When *HKR1* was first isolated, a Northern analysis detected a short mRNA dominantly in those transformants harboring *HKR1*^tr^, while only a long mRNA (about 6 kb in length) was detected in the parental strain [4]. Although the exonic promoter driving *HKR1*^tr^ showed transcriptional activity when present on a plasmid, it remained unclear whether *HKR1*^tr^ transcription might be repressed by the sequence further upstream of the presumptive transcription start sites identified above. The reporter constructs carrying the 412-bp sequence upstream of the ATG codon corresponding to Met-1137 (sequence 3 in Fig 4A) produced stronger promoter activity than that obtained with the 160-bp exonic promoter fragment (Fig 4B and 4C). Surprisingly, extending the length of the exonic sequence cloned upstream of the reporter genes to include 795 bp upstream of the ATG codon (sequence 4, Fig 4A) yielded clearly weaker fluorescence emitted by the transformants (Fig 4B) and lower β-galactosidase activity (Fig 4C) than did the 412-bp sequence. Furthermore, using the 2,502 bp-*HKR1* coding sequence and the entire 3,408-bp *HKR1* coding sequence from the original ATG codon to the ATG corresponding to Met-1137 (sequences 5 and 6, Fig 4A) abolished fluorescence (Fig 4B) and β-galactosidase activity (Fig 4C). This change in reporter gene output was not a consequence of copy number variation among the plasmids tested, as determined by quantitative PCR (S2 Fig). The above results indicate that *HKR1*^tr^ transcription is repressed by the upstream sequence within the longer *HKR1* exon, and we conclude that the sequence from the original ATG codon to around the nucleotide position #3000 (Region B in Fig 4A) contains one or more elements that negatively regulate the transcriptional activity of the exonic promoter.

**Fig 5:**
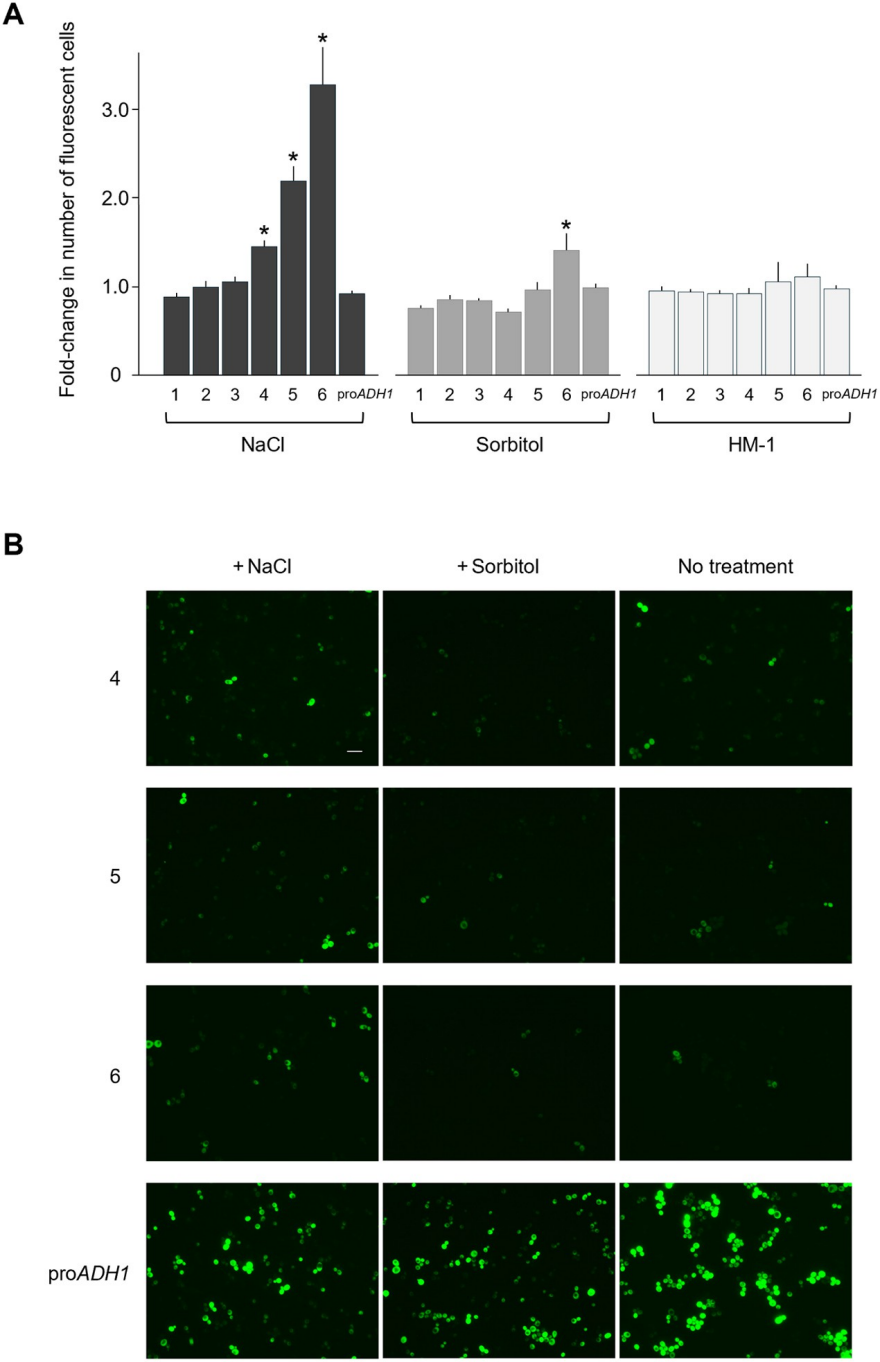
Effects of NaCl, sorbitol, or the HM-1 killer toxin on the expression of the reporter gene *mUkG1* driven by the exonic *HKR1* promoter. **(A)** Fold-changes in the number of fluorescent-positive cells harboring the constructs illustrated in Fig 4A cultured with NaCl, sorbitol, or HM-1, as determined by flow cytometry. Each construct was introduced into *S*. *cerevisiae* A451 cells, and the transformants were grown under control growth conditions or cultured with 250 mM NaCl for 18 h. The number of fluorescent cells upon NaCl treatment among all transformants was normalized to that under control conditions. In addition, the same experiment was carried out with 1 M sorbitol or the 50% lethal dose of HM-1 treatment. The *mUkG1* reporter gene driven by the *ADH1* promoter (pro*ADH1*) was used as a control. Six independent primary transformants were tested for each construct. Values are means with standard deviation. * *p* < 0.01 based on the paired samples t-test. **(B)** Representative fluorescence microscope photographs of cells carrying the *mUkG1* gene driven by the exonic *HKR1* promoter sequence 4, 5, or 6 treated with 250 mM NaCl (+NaCl), 1 M sorbitol (+Sorbitol), or maintained under control conditions (No treatment). The *ADH1* promoter (pro*ADH1*) was used as a control. The scale bar indicates a length of 10 μm.

We next wondered whether growth conditions might affect this transcriptional repression. Hkr1 has been extensively studied as a component of the HOG pathway [1–3]. We therefore tested mUkG1 fluorescence in cells exposed to a hyperosmolar stimulus by the addition of 250 mM NaCl or 1 M sorbitol using flow cytometry. We also observed the fluorescent cells carrying the various constructs shown in Fig 4A by fluorescence microscopy. When *mUkG1* was cloned downstream of the region from the nucleotide position #1 to #3408 of *HKR1* (sequence 6 in Fig 4A), few transformants displayed fluorescence under normal growth conditions. Following treatment with 250 mM NaCl, however, to create a higher osmotic state, the number of fluorescent cells increased 3.3-fold compared to the control (2.2% to 7.2% of total cells) as shown in Fig 5A and Sheet #4 of S1 File. Furthermore, the number of mUkG1 positive cells was also increased using the region from the nucleotide position #907 to #3408 of *HKR1* (sequence 5, Fig 4A) with a 2.2-fold rise (6.6% to 14.6%), shown in Fig 5A and Sheet #4 of S1 File, and a similar, albeit weaker, effect was observed when *mUkG1* was cloned downstream of the region from the nucleotide position #2614 to #3408 of *HKR1* (sequence 4, Fig 4A), with a 1.5-fold rise (18.1% to 26.3%) as shown in Fig 5A and Sheet #4 of S1 File. Treatment with 1 M sorbitol also resulted in a similar change in the number of fluorescent cells when sequence 6 was used, but only by 1.4-fold (from 2.2% to 3.0%), shown in Fig 5A and Sheet #5 of S1 Fle. Plasmid copy number was not responsible for the observed changes in fluorescence between the groups, as determined by quantitative PCR (S2 Fig). We confirmed these results by direct observation under a fluorescence microscope (Fig 5B). For the clones harboring shorter sequences upstream of the ATG codon corresponding to Met-1137 (sequences 1, 2, and 3), we observed no significant increase in the number of fluorescent cells upon exposure to NaCl or sorbitol.

The addition of NaCl at a concentration of 250 mM creates a relatively mild hyperosmotic state. We also tested higher concentrations (up to 500 mM) of NaCl treatment and resulted in a similar change in the expression of the reporter mUkG1, but these higher osmotic conditions severely affected the growth of the cells.

In addition, we investigated expression of the reporter driven by the exonic *HKR1* promoter sequences (sequences 1–6 in Fig 4A) under the influence of HM-1 at a concentration resulting in 50% cell death in wild-type cells, but we observed no significant changes (Fig 5A). Hkr1 was first identified as a factor conferring resistance to HM-1 before it was found to function in the HOG pathway [4]. However, these results show that the exonic *HKR1* promoter does not respond to stimulation by the addition of HM-1.

## Discussion

The *HKR1* exon exhibits an extremely unusual phenomenon: it has promoter activity while also encoding a protein sequence. A full-length *HKR1* transcript is transcribed from the original promoter located at the 5′ end to produce full-length Hkr1, while an internal transcription start site drives the transcription of a truncated *HKR1* transcript encoding a shorter C-terminal Hkr1 fragment. Thus, one gene can produce one large, full-size protein and a small, truncated variant. In this study, we mapped the regulatory regions of this exonic promoter and quantified its activity using a fluorescent protein and β-galactosidase as reporters (Figs 3 and 4). We also verified the functionality of the truncated protein encoded by the resulting shorter transcript (S1B Fig and Fig 2). Interestingly, this exonic promoter was not preferred over the promoter producing the full-length transcript under normal culture conditions, but we observed a tendency for the repressive transcriptional regulation to be alleviated by the addition of NaCl or sorbitol to the culture (Fig 5 and Sheets #4 and #5 of S1 File). The choice of promoter and transcription start site would therefore be another means to diversify the transcriptome and proteome as reported in plants and insects [29, 30], in addition to alternative splicing.

While transcription generally starts at the 5′ end of the first exon, in very rare cases exonic sequences display promoter activity and produce a 5′-truncated transcript from the same exon as the full-length gene that encodes a functional protein. One such example is an exonic promoter in the voltage-gated calcium channel gene *CACNA1C*, expressed in murine neural cell line Neuro2A. The shorter *CACNA1C* transcript is initiated from a promoter within exon 46 and encodes calcium channel-associated transcriptional regulator CCAT, which acts as a transcription factor that regulates neurite extension and inhibits *CACNA1C* expression [31]. In another example from *S*. *cerevisiae*, an internal promoter within the vacuolar iron transporter gene cross-complements Ca^2+^ phenotype of *csg1* (*CCC1*) coding region produces a truncated *CCC1* transcript that encodes a protein still conferring protection from iron toxicity [32]. Although in mammalian systems there are a few other examples of promoter or promoter-like sequences found in exons of genes that have introns [33, 34], a functional promoter sequence within an intronless gene is extremely rare.

Although we did not identify typical TATA boxes within the exonic promoter sequence, we did notice scattered TATA-like sequences. Many *S*. *cerevisiae* genes lack a TATA box, as does the original *HKR1* promoter. To define the transcription start site(s), we performed a 5′-RACE assay, which revealed that the transcription start site might shift depending on the length of the promoter fragment harbored by the transformants used for the 5′-RACE assay (S3 Fig). These results may reflect the plasmid-based aspect of this experiment, in which the transcription start sites were examined for a specific length of the exonic promoter.

**Fig 6:**
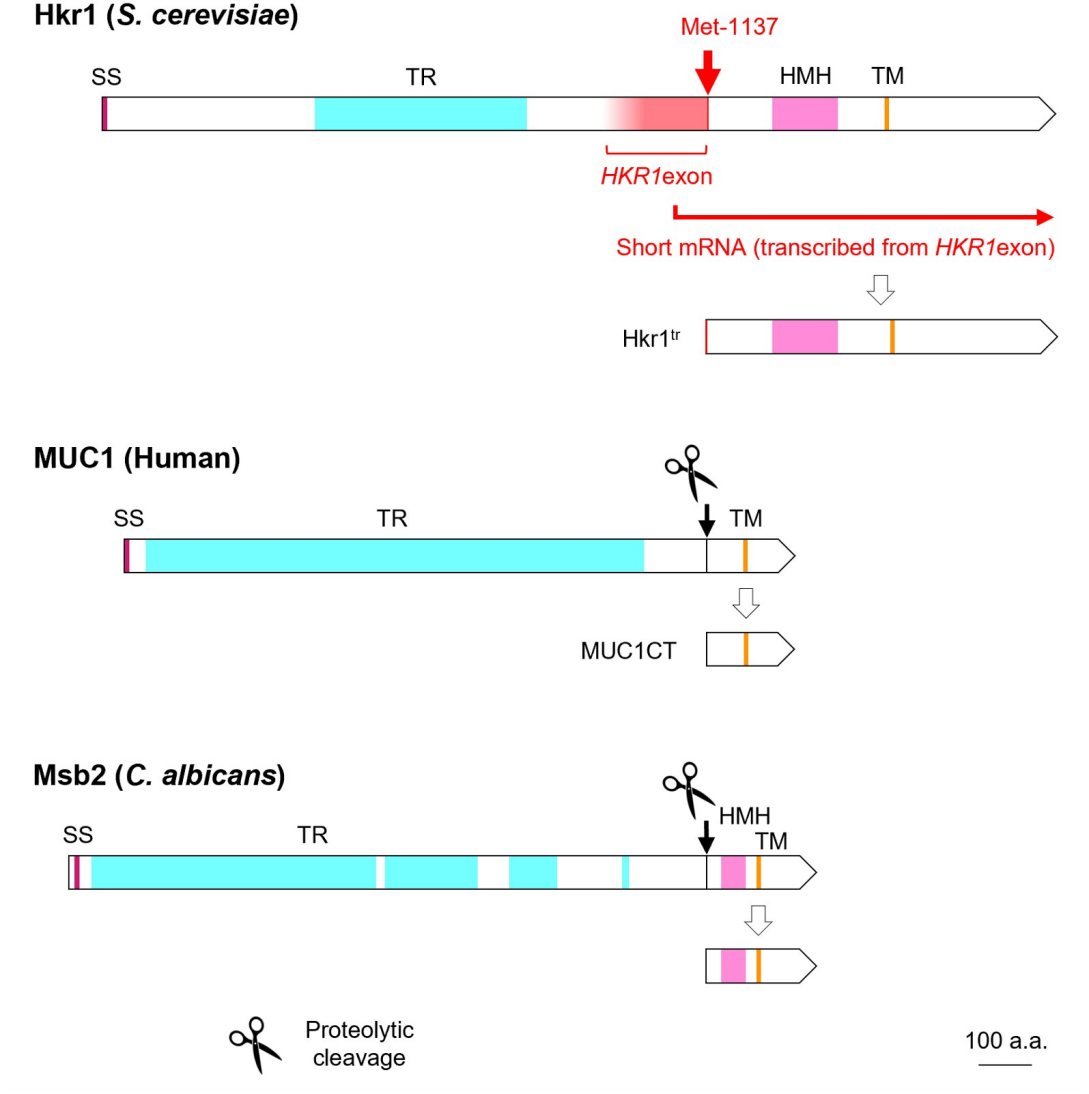
Structural comparison between *S*. *cerevisiae* Hkr1^tr^ produced from the exonic *HKR1* promoter and the fragments generated after proteolytic processing of human MUC1 and *C*. *albicans* Msb2. Diagrams of *S*. *cerevisiae* Hkr1, human MUC1, and *C*. *albicans* Msb2. Met-1137 of Hkr1 may serve as an internal translation initiation site following transcription from the exonic *HKR1* promoter (*HKR1*exon) to produce the C-terminal portion of the protein, Hkr1^tr^, which is assumed to participate as a signal transduction messenger. Proteolytic cleavage of MUC1 and Msb2 produces short C-terminal fragments. For the domain names, please see Fig 1.

The exonic *HKR1* promoter may help the transduction efficiency of this signaling molecule within its pathway(s). One possible mechanism would be the production of variants with different combinations of functional domains. Full-length human MUC1, for example, is cleaved into its extracellular domain and its cytoplasmic tail (MUC1CT) through processing, after which MUC1CT retaining the transmembrane domain translocates to the cytoplasm and eventually to the nucleus, where it completes its function as a signal messenger by affecting transcription of downstream genes [15, 21, 35, 36]. Likewise, Msb2 of *S*. *cerevisiae* is subjected to proteolytic cleavage by the aspartyl protease yapsin 1 [20]; Msb2 of *Candida albicans* is also processed in a similar manner [37]. It is unclear whether Hkr1 undergoes such proteolytic processing, it can be noted, however, that Hkr1^tr^ shares some structural similarity with MUC1CT and the C-terminal fragment of Msb2, such as the presence of a transmembrane domain in the middle (Fig 6). Regarding subcellular localization and behavior of Hkr1^tr^, it has not been confirmed whether it migrates to the nucleus, but Tatebayashi *et al*. [1] and Yabe *et al*. [5] reported that it was located inside the cell under certain conditions. Furthermore, since Hkr1^tr^ has a leucine zipper motif, it is structurally possible that it interacts with DNA in the nucleus and is involved in regulating gene expression. Although Hkr1, MUC1, and Msb2 are all known to be transmembrane proteins, their C-terminal portions may share the common property of translocating from the plasma membrane to the cytoplasm, and then to the nucleus to function [15, 21, 35, 38].

One of characteristic sequences contained in sequences 5 and 6 in Fig 4A that leads to a repression of transcriptional activity from the exonic promoter is the tandem repeat; repetitive DNA sequences may cause gene silencing [39, 40]. There are also reports that short tandem repeats are involved in the regulation of gene expression in eukaryotes [41, 42]. Considering these previous research findings, we can speculate that the sequence encoding the tandem repeat of Hkr1 may not only downregulate the transcriptional activity of the exonic promoter, but also be involved in positive regulation. Although our results did not show such clear changes to support this hypothesis directly, it is possible that the region containing the repetitive sequence (Region B in Fig 4A) may be the cause of the slight alleviation of the transcriptional repression (Fig 5 and Sheets #4 and #5 of S1 File). A tandem repeat is a commonly observed structure among mucin family proteins, including Hkr1, suggesting that this tandem repeat might be involved in expressional regulation beyond *HKR1*.

It would be interesting if the transcriptional activity of the repressed exonic promoter is restored by osmotic stimulation. In our experimental data in which osmotic stress was applied by adding NaCl, the percentage of fluorescent cells increased 3.3-fold (from 2.2% to 7.2%) with the longest exonic sequence (sequence 6 in Fig 4A) as shown in Fig 5A and Sheet #4 of S1 File. Similarly, when shorter sequence 5 was used, the percentage increased 2.2-fold (from 6.6% to 14.6%), and with sequence 4, the percentage increased 1.5-fold (from 18.1% to 26.3%). When osmotic stress was applied by sorbitol, the percentage increased only 1.4-fold (from 2.2% to 3.0%) with sequence 6 (Fig 5A and Sheet #5 of S1 File). It was difficult to confirm a significant increase with other sequences (Fig 5A and Sheets #4 and #5 of S1 File). Although these values are too small to be considered as significant activation of transcription, the values change depending on the length of the exonic sequence used in the assay, so it is conceivable that this phenomenon is caused by some sequence element(s) contained in the region. The main reason for the small number of fluorescent cells is presumably that the transcription level of *HKR1*^tr^ is extremely low, as it is thoroughly repressed by its upstream sequence. It can be considered that since its intrinsic expression level is low, even a slight change in transcription may have biological and physiological significance.

Regarding the relationship between the exonic *HKR1* promoter and HM-1 resistance, HM-1 did not affect the activity of the exonic promoter under our experimental conditions (Fig 5A and Sheet #6 of S1 File). We therefore infer that the acquisition of HM-1 resistance by overexpressing Hkr1^tr^ is perhaps a sideshow function of Hkr1. It is known that the killer activity of HM-1 is affected by external factors. Komiyama *et al*. reported that the addition of NaCl to the culture decreased the cytocidal effect of HM-1 [43]. Since HM-1 is a basic and cationic protein with an isoelectric point at 9.1 that tends to be adsorbed to negatively charged yeast cells [44], addition of electrolytes such as NaCl may neutralize the charge and eliminate the electrical binding affinity resulting in a weakening of its killer activity, but on the other hand, addition of non-electrolyte sorbitol reduces killer activity, too [43]. The transcriptional activation of the exonic promoter by external factors remains unclear at present, but it may still be worth discussing in more detail the relationship between factors that stimulate the transcriptional activity of the exonic promoter and the acquisition of resistance to HM-1.

We performed all of our experiments using a plasmid-based system; these results will need to be confirmed for the endogenous *S*. *cerevisiae* gene. However, *HKR1* is transcribed at an extremely low level [4], as is *HKR1*^tr^, which contributes to the difficulty in quantitatively evaluating the two *HKR1* promoters in the genome context.

The number of functional genes is estimated to be around 6,000 in *S*. *cerevisiae* [45], and around 20,000 in humans [46]. These are not small numbers by any means, but are still limited. If a single gene is expressed in different ways through heterogeneous transcription from distinct promoters, it will lead to diversification of expression patterns and a greater variety of biological activities from a limited number of genes. This finding may become another example of such a phenomenon.

## Conclusions

*S*. *cerevisiae* cells exhibited resistance to HM-1 upon the introduction of an apparently promoterless 3′ portion of *HKR1* (*HKR1*^tr^). We detected promoter activity from the *HKR1* exon (exonic promoter) and mapped the responsible region to the sequence between the nucleotide positions #3000 and #3408 downstream of the original ATG. We showed that the ATG codon corresponding to Met-1137 of Hkr1 is used as an internal translation initiation site for the truncated form of Hkr1 (Hkr1^tr^). The sequence upstream of the exonic promoter within the same exon is involved in transcriptional regulation, especially in the repressive regulation of *HKR1*^tr^.

## Supporting information

S1 Fig Determination of the minimal region responsible for HM-1 killer toxin resistance. (A) The deletions of *HKR1* (sequences I–IV) were subcloned into the vector pYES2 downstream of the *GAL1* promoter (pro*GAL1*) for the overexpression in *S*. *cerevisiae* (constructs I–IV). (B) Proteins that could be produced from the *HKR1* deletions are illustrated (I–IV). The resistance of each transformant to HM-1 was tested, as indicated to the right.
(TIF)

S2 Fig Quantitative PCR for the evaluation of copy number stability of the plasmids. The copy numbers of the plasmids in which *mUkG1* was ligated downstream sequence 1, 2, 3, 4, 5, or 6 in Fig 4A were examined by qPCR. The relative copy numbers of the plasmids carrying the sequences 1–6 to those carrying only the *mUkG1* coding sequence are shown (No treatment). The values of the transformed cells treated with 250 mM NaCl (+NaCl) or 1 M sorbitol (+Sorbitol) are shown relative to the same transformants cultured with no addition of NaCl or sorbitol. Three independent primary colonies were picked up for each construct to extract DNA and the quantifying reactions were performed in triplicate. Values are means with standard deviation. The Tukey test revealed that no significant differences were observed between any of the samples under each culture condition.
(TIF)

S3 Fig Putative transcription start sites in the exonic *HKR1* promoter. Transcription start sites in the exonic promoter were determined by 5′-RACE and are shown as wedges. Transcription started at the nucleotide position #3075, #3361, or #3383 into the *HKR1* sequence depending on the clone sequenced. The nucleotide sequence is shown below the schematic diagrams of *HKR1*. For the domains, Regions 1 and 2, please see Figs 1 and 3B.
(TIF)

S1 Table Primers used in this study. (DOCX)

S2 Table Plasmids used in this study. (DOCX)

S1 File Original data used to create Figs 3B, 4C and 5A, and S1 and S2 Figs. (XLSX)
