# Supplementary figures and images for: A cryptic promoter in the exon of HKR1 drives expression of a truncated form of Hkr1 in Saccharomyces cerevisiae
Source: PLoS One. 2024 Nov 21;19(11):e0314016. doi: 10.1371/journal.pone.0314016 (PMC11581313; doi:10.1371/journal.pone.0314016)

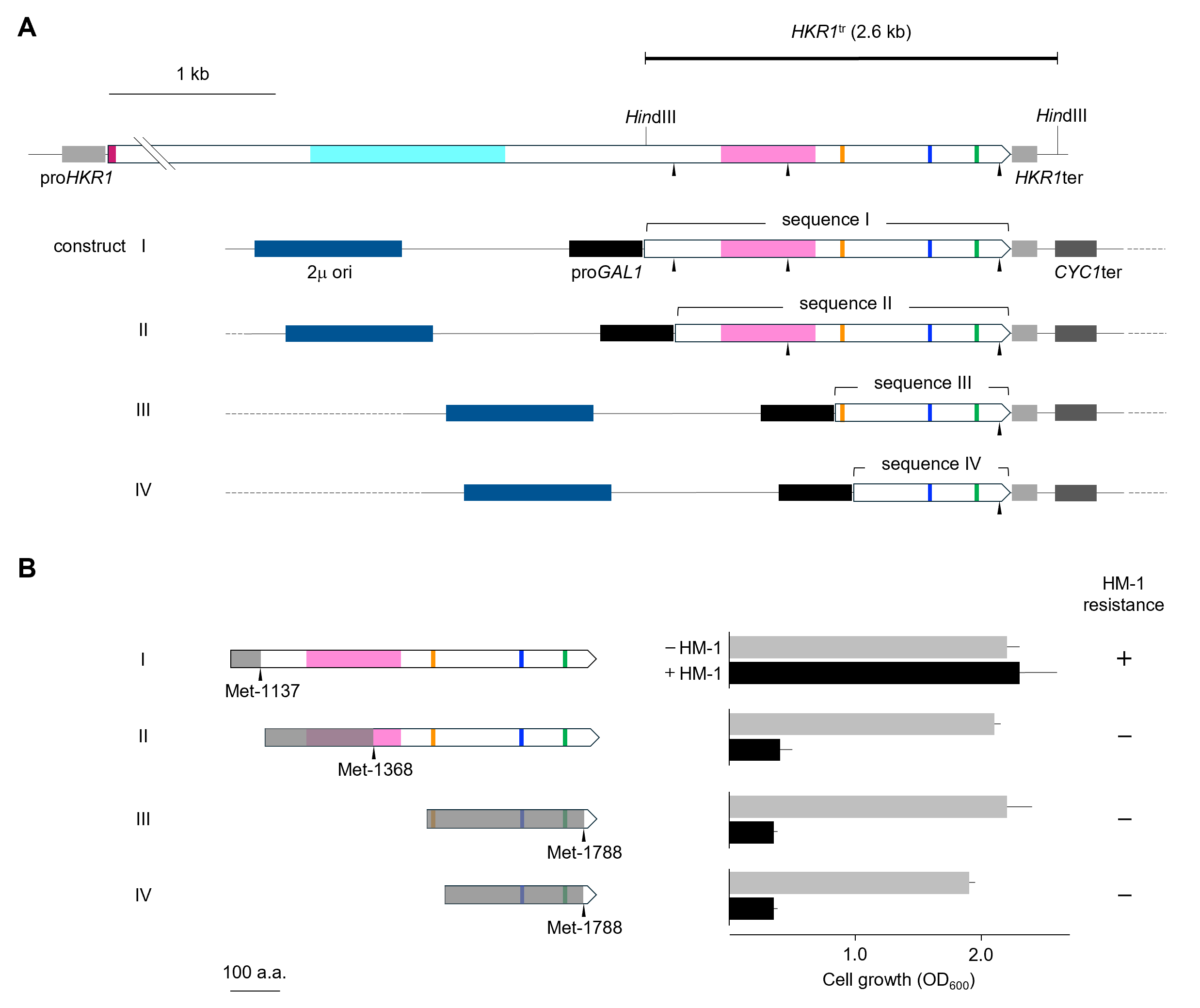

Supplement: S1 Fig — (A) The deletions of HKR1 (sequences I–IV) were subcloned into the vector pYES2 downstream of the GAL1 promoter (proGAL1) for the overexpression in S. cerevisiae (constructs I–IV). (B) Proteins that could be produced from the HKR1 deletions are illustrated (I–IV). The resistance of each transformant to HM-1 was tested, as indicated to the right. (TIF) [file pone.0314016.s001.tif]

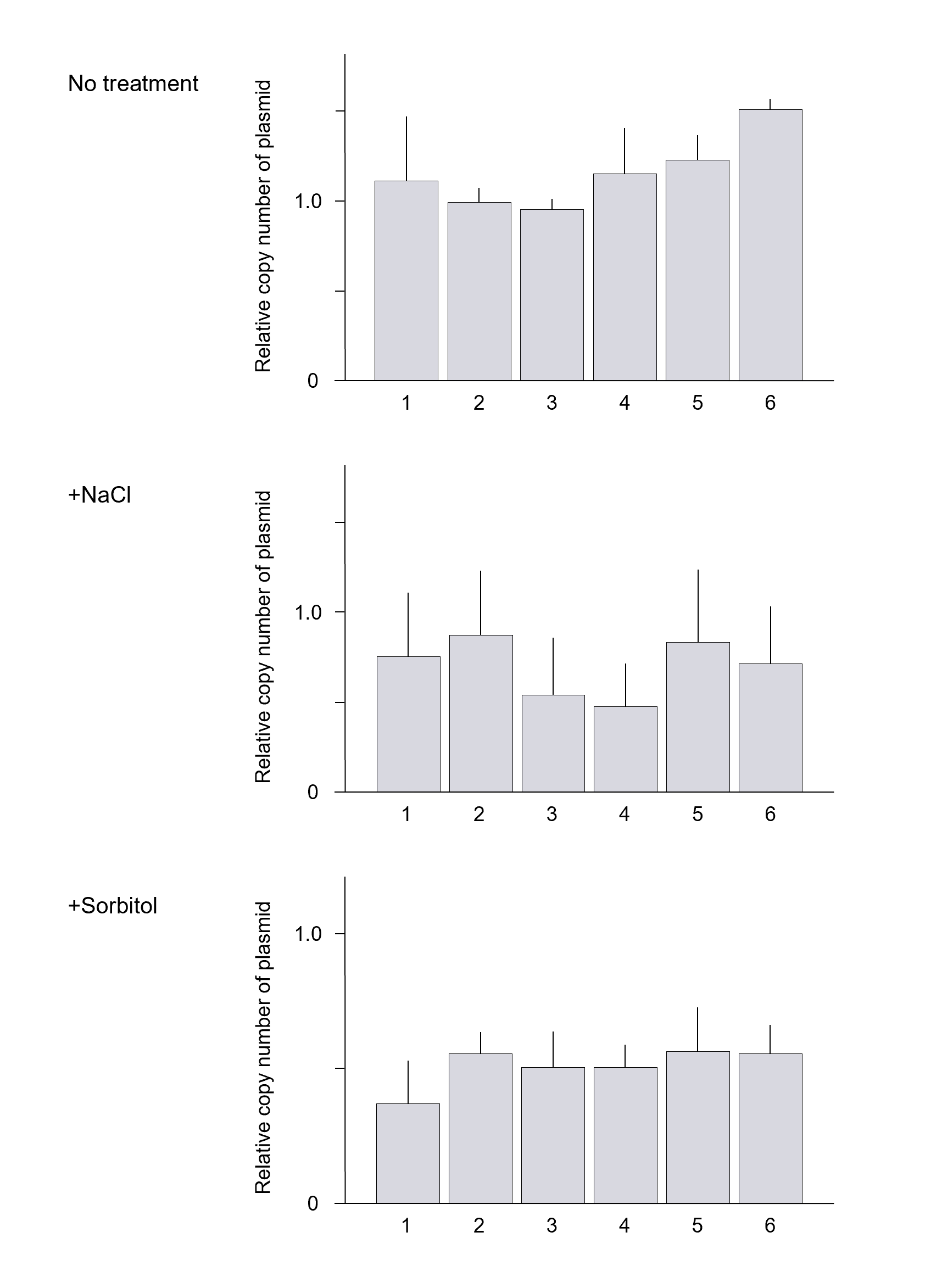

Supplement: S2 Fig — The copy numbers of the plasmids in which mUkG1 was ligated downstream sequence 1, 2, 3, 4, 5, or 6 in Fig 4A were examined by qPCR. The relative copy numbers of the plasmids carrying the sequences 1–6 to those carrying only the mUkG1 coding sequence are shown (No treatment). The values of the transformed cells treated with 250 mM NaCl (+NaCl) or 1 M sorbitol (+Sorbitol) are shown relative to the same transformants cultured with no addition of NaCl or sorbitol. Three independent primary colonies were picked up for each construct to extract DNA and the quantifying reactions were performed in triplicate. Values are means with standard deviation. The Tukey test revealed that no significant differences were observed between any of the samples under each culture condition. (TIF) [file pone.0314016.s002.tif]

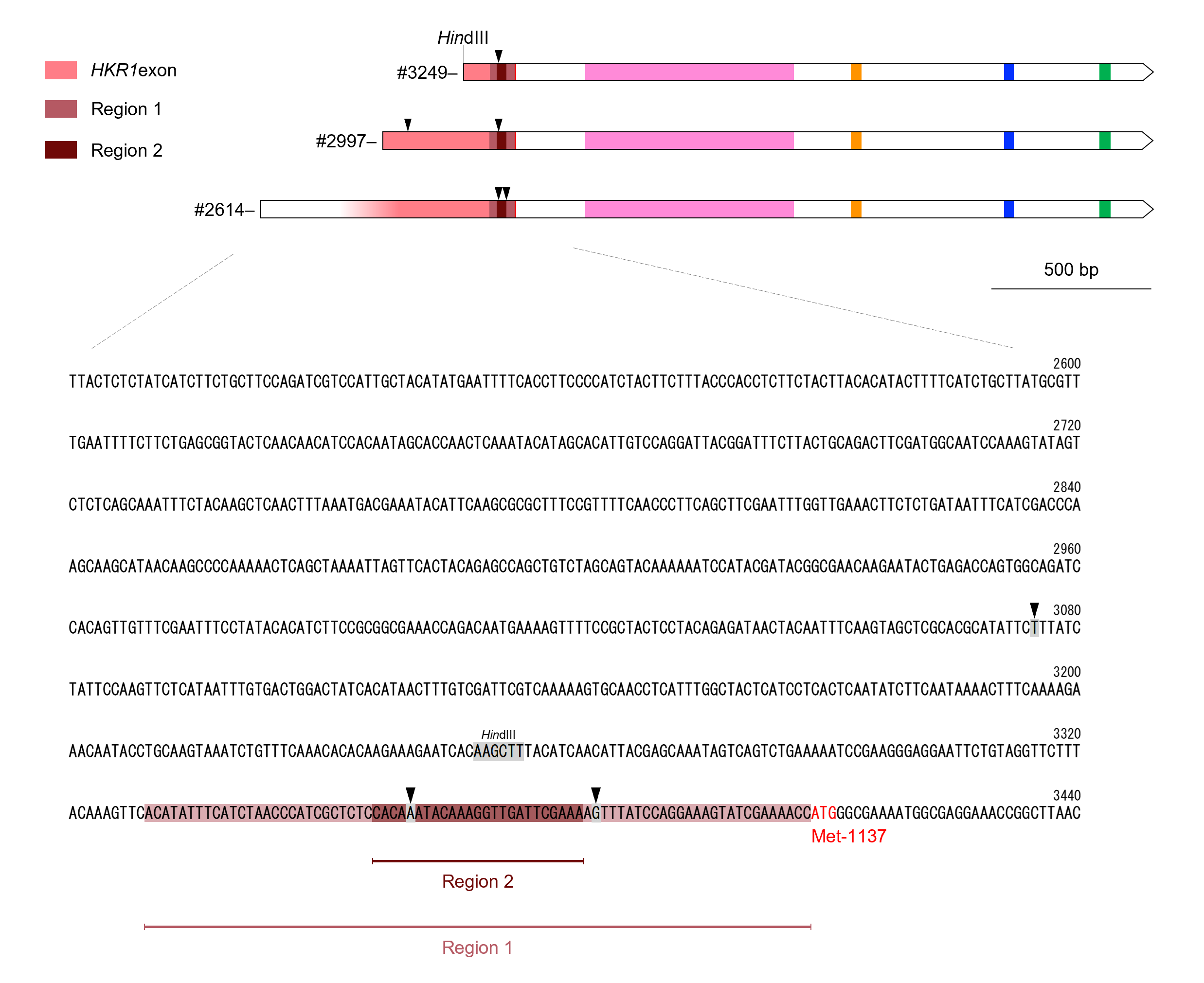

Supplement: S3 Fig — Transcription start sites in the exonic promoter were determined by 5′-RACE and are shown as wedges. Transcription started at the nucleotide position #3075, #3361, or #3383 into the HKR1 sequence depending on the clone sequenced. The nucleotide sequence is shown below the schematic diagrams of HKR1. For the domains, Regions 1 and 2, please see Figs 1 and 3B. (TIF) [file pone.0314016.s003.tif]
