# Supplementary material for: A cryptic promoter in the exon of HKR1 drives expression of a truncated form of Hkr1 in Saccharomyces cerevisiae
Source: PLoS One. 2024 Nov 21;19(11):e0314016. doi: 10.1371/journal.pone.0314016 (PMC11581313; doi:10.1371/journal.pone.0314016)
Supplement: S1 Table — (DOCX) [file pone.0314016.s004.docx]

**S1 Table Primers used in this study.**

| Primer | Position in *HKR1* | Orientation | Sequence | Length  (mer) | Description |
| --- | --- | --- | --- | --- | --- |
| HKR1-Fw1  HKR1-Fw2  HKR1-Fw3  HKR1-Fw4  HKR1-Fw5a  HKR1-Fw5b  HKR1-Fw5c  HKR1-Fw6  HKR1-Fw7  HKR1-Fw8  HKR1-Fw9  HKR1-Fw10  HKR1-Fw11  HKR1-Fw12  HKR1-Fw13  HKR1ΔM-1137-Fw  HKR1q-Fw  lacZ-Fw1  mUkG1-Fw1  HKR1-Rv1  HKR1-Rv2  HKR1-Rv3  HKR1-Rv4a  HKR1-Rv4b  HKR1-Rv4c  HKR1-Rv4d  HKR1ΔM-1137-Rv  HKR1q-Rv  HKR1RT-Rv  mUkG1-Rv1 | #1  #907  #2614  #2997  #3232  #3247  #3249  #3276  #3303  #3330  #3357  #3382  #4048  #4909  #5383  #3409  #3780       #3326  #4087  #4950  3'-noncoding  3'-noncoding  3'-noncoding  3'-noncoding  #3427  #3898  #3895 | Forward  Forward  Forward  Forward  Forward  Forward  Forward  Forward  Forward  Forward  Forward  Forward  Forward  Forward  Forward  Forward  Forward  Forward  Forward  Reverse  Reverse  Reverse  Reverse  Reverse  Reverse  Reverse  Reverse  Reverse  Reverse  Reverse | CCCCTCGAGTATAAAGATGGTCTCATTG  CCCCTCGAGTGGCTGTTGGAGTATCCC  CCCCTCGAGCGGTACTCAACAACATCC  GGGCTCGAGGGCGAAACCAGACAATGAAA  CCCCCATATGACACAAGAAAGAATCACAAGC  AAAGGTCGACAAGCTTTACATCAACATTAC  AAAACTCGAGAAGCTTTACATCAACATTAC  AAAACTCGAGTAGTCAGTCTGAAAAATC  AAAACTCGAGAATTCTGTAGGTTCTTTAC  AAAACTCGAGACATATTTCATCTAACCC  AAAACTCGAGCACAAATACAAAGGTTG  GGGGCTCGAGTTTATCCAGGAAAGTATCG  TTACATCCTCCACAAGTC  GAGGACTCCATATCAGGC  AAACTGCAGAACCATCAAACGACA  TAGGGCGAAAATGGCGAGGAAACC  CACGGCCGCTTTGAATTATGT  AAAACTCGAGATGGCCATGATTACGGATTC  GGCTCTGTTGCTTGAAGGAG  CTTTGTAAAGAACCTACAG  TCACCGCTATTGAGGATG  ACCATTTTCATCAACATC  AAAAAAGCTTGGTAGGTTCTTCTCCG  AAAGTCGACAAGCTTGGTAGGTTCTTC  AAAACTCGAGCTTGGTAGGTTCTTCTCCG  AAACTCGAGAAGCTTGGTAGGTTCTTCTC  CCTCGCCATTTTCGCCCTAGGTTTTCGATACTTTCC  TGTCCAGTTCTGATGAGGTGT  CCAGTTCTGATGAGG  TACGATGGTCGATCTTGTGG | 28  27  27  29  31  30  30  28  29  28  27  29  18  18  24  24  21  30  20  19  18  18  26  27  29  29  36  21  15  20 | *Xho*I  *Xho*I  *Xho*I  *Xho*I  *Nde*I  *Sal*I  *Xho*I  *Xho*I  *Xho*I  *Xho*I  *Xho*I  *Xho*I  *Pst*I  for mutagenesis  for qPCR^*^  *Xho*I, *lacZ*-coding  *mUkG1*-coding, for qPCR  *Hin*dIII  *Sal*I-*Hin*dIII  *Xho*I  *Xho*I-*Hin*dIII  for mutagenesis  for qPCR  for 5′-RACE^**^, phosphorylated  *mUkG1*-coding, for qPCR |

^*^qPCR quantitative PCR

^**^5′-RACE 5′ rapid amplification of cDNA ends
