# Supplementary material for: A cryptic promoter in the exon of HKR1 drives expression of a truncated form of Hkr1 in Saccharomyces cerevisiae
Source: PLoS One. 2024 Nov 21;19(11):e0314016. doi: 10.1371/journal.pone.0314016 (PMC11581313; doi:10.1371/journal.pone.0314016)
Supplement: S2 Table — (DOCX) [file pone.0314016.s005.docx]

**S2 Table Plasmids used in this study.**

| Plasmid | Gene, replication origin, promoter, and terminator | Description |
| --- | --- | --- |
| pYES-HKR1^tr^ I  pYES-HKR1^tr^ II  pYES-HKR1^tr^ III  pYES-HKR1^tr^ IV  pYEHKR1^tr^-Fw  pYEHKR1^tr^-Rv  pYEHKR1^tr^-Rv-ΔMet-1137  pYE-exon160UkG1  pYE-UkG1  pYE-ADHUkG1  pYC-exon160-lacZ  pYC-exon133-lacZ  pYC-exon106-lacZ  pYC-exon79-lacZ  pYC-exon52-lacZ  pYC-exon27-lacZ  pYE-exon52-UkG1  pYE-exon412-UkG1  pYE-exon795-UkG1  pYE-exon2502-UkG1  pYE-exon3408-UkG1  pYC-lacZ  pYC-exon412-lacZ  pYC-exon795-lacZ  pYC-exon2502-lacZ  pYC-exon3408-lacZ  pYEHKR1-Rv-3249  pYEHKR1-Rv-2997  pYEHKR1-Rv-2614 | 2 μ / Amp / *URA3* / f1 / pro*GAL1*-*HKR1*(3249-term)-*CYC1*ter  2 μ / Amp / *URA3* / f1 / pro*GAL1*-*HKR1*(3412-term)-*CYC1*ter  2 μ / Amp / *URA3* / f1 / pro*GAL1*-*HKR1*(4441-term)-*CYC1*ter  2 μ / Amp / *URA3* / f1 / pro*GAL1*-*HKR1*(4525-term)-*CYC1*ter  2 μ / Amp / *LEU2* / *HKR1*^tr^ (forward direction with *LEU2*)  2 μ / Amp / *LEU2* / *HKR1*^tr^ (opposite direction to *LEU2*)  2 μ / Amp / *LEU2* / *HKR1*^tr^ with ΔMet-1137 mutation  2 μ / Amp / *LEU2* / *HKR1*(3249-3408)-*mUkG1*-*HKR1*ter  2 μ / Amp, *LEU2* / *mUkG1*-*HKR1*ter  2 μ / Amp / *LEU2* / pro*ADH1*-*mUkG1*-*ADH1*ter  ARS1-CEN4 / Amp / *URA3* / *HKR1*(3249-3408)-*lacZ*-*HKR1*ter  ARS1-CEN4 / Amp / *URA3* / *HKR1*(3276-3408)-*lacZ*-*HKR1*ter  ARS1-CEN4 / Amp / *URA3* / *HKR1*(3303-3408)-*lacZ*-*HKR1*ter  ARS1-CEN4 / Amp / *URA3* / *HKR1*(3330-3408)-*lacZ*-*HKR1*ter  ARS1-CEN4 / Amp / *URA3* / *HKR1*(3357-3408)-*lacZ*-*HKR1*ter  ARS1-CEN4 / Amp / *URA3* / *HKR1*(3382-3408)-*lacZ*-*HKR1*ter  2 μ / Amp / *LEU2* / *HKR1*(3357-3408)-*mUkG1*-*HKR1*ter  2 μ / Amp / *LEU2* / *HKR1*(2997-3408)-*mUkG1*-*HKR1*ter  2 μ / Amp / *LEU2* / *HKR1*(2614-3408)-*mUkG1*-*HKR1*ter  2 μ / Amp / *LEU2* / *HKR1*(907-3408)-*mUkG1*-*HKR1*ter  2 μ / Amp / *LEU2* / *HKR1*(1-3408)-*mUkG1*-*HKR1*ter  ARS1-CEN4 / Amp / *URA3* / *lacZ*-*HKR1*ter  ARS1-CEN4 / Amp / *URA3* / *HKR1*(2997-3408)-*HKR1*ter  ARS1-CEN4 / Amp / *URA3* / *HKR1*(2614-3408)-*HKR1*ter  ARS1-CEN4 / Amp / *URA3* / *HKR1*(907-3408)-*HKR1*ter  ARS1-CEN4 / Amp / *URA3* / *HKR1*(1-3408)-*HKR1*ter  2 μ / Amp / *LEU2* / *HKR1* (3249-term, opposite direction to *LEU2*)  2 μ / Amp / *LEU2* / *HKR1* (2997-term, opposite direction to *LEU2*)  2 μ / Amp / *LEU2* / *HKR1* (2614-term, opposite direction to *LEU2*) | pYES2 backbone, construct I in S1 Fig  pYES2 backbone, construct II in S1 Fig  pYES2 backbone, construct III in S1 Fig  pYES2 backbone, construct IV in S1 Fig  Fig 2  Fig 2  Fig 2  Fig 3A, sequence 2 in Fig 4  Figs 3A and 4A, promoterless negative control  Fig 3A, positive control driven by pro*ADH1*  pAUR112 backbone, Fig 3B, sequence 2 in Fig 4  pAUR112 backbone, Fig 3B  pAUR112 backbone, Fig 3B  pAUR112 backbone, Fig 3B  pAUR112 backbone, Fig 3B, sequence 1 in Fig 4  pAUR112 backbone, Fig 3B  sequence 1 in Fig 4  sequence 3 in Fig 4  sequence 4 in Fig 4  sequence 5 in Fig 4  sequence 6 in Fig 4  pAUR112 backbone, Fig 4,  promoterless negative control  pAUR112 backbone, sequence 3 in Fig 4  pAUR112 backbone, sequence 4 in Fig 4  pAUR112 backbone, sequence 5 in Fig 4  pAUR112 backbone, sequence 6 in Fig 4  = pYEHKR1^tr^-Rv, for 5′-RACE, S3 Fig  for 5′-RACE, S3 Fig  for 5′-RACE, S3 Fig |

Amp ampicillin resistance gene of *E. coli*

ARS1 autonomously replicating sequence of *S. cerevisiae*

CEN4 centromeric sequence of *S. cerevisiae* chromosome IV

*HKR1*^tr^ 2.6-kb truncated form of *HKR1* with terminator

*HKR1*(xxx–yyy) partial sequence of *HKR1* ranging nucleotide position #xxx through #yyy

*lacZ* reporter β-galactosidase gene from *E. coli*

*LEU2* auxotrophic marker gene of *S. cerevisiae* for leucine synthesis

*mUkG1* reporter fluorescent protein gene *mUkG1* (monomeric Umikinoko-Green 1) from *Sarcophyton* sp.

pro*ADH1* promoter of *S. cerevisiae* *ADH1*

pro*GAL1* promoter of *S. cerevisiae* *GAL1*

*ADH1*ter terminator of *S. cerevisiae* *ADH1*

*CYC1*ter terminator of *S. cerevisiae* *CYC1*

*HKR1*ter terminator of *S. cerevisiae* *HKR1*

*URA3* auxotrophic marker gene of *S. cerevisiae* for uracil synthesis

2 μ replication origin of *S. cerevisiae* 2-micron DNA
